# 

**Published:** 1906-01-15

**Authors:** 


### SUBJECT INDEX TO VOLUME LX.

### EVENT AND COMMENT

Amendments to Army and Navy Dental Bills, 111.

Army and Navy Dental Bills, 109.

Color Problem, 113.

Columbus Dental Library, 595.

Corrections, 492.

Death from Gum Infection, 379.

Editor Gone Wrong, 218.

Independent Practitioners, 56.

International Journal Discontinues Publication, 56.

Literary versus Clinical Program, 163.

Medical Education in Indiana, 219.

National Dental Association Clinic, 166.

National Meetings, 381.

New Form of Swindle, 114.

Ohio State Dental Society, 596.

Plea for Improved Curriculum, 541

Porto Rico Dental Law, 55.

Professional Man or Mechanic, 165.

Professional Men and Business Affairs, 327.

Professor W. D. Miller, 273.

Prophylaxis, 271.

Prophylaxis Defined, 487.

Proprietary Medicines, 219.

Pulpless Teeth and Pyorrhoea, 217.

San Francisco Situation, 325.

Temporary Licenses, 220.

Thymocamphene, 434.

Thymophene, 433.

What We Seem and What We Are, 434.

When in Doubt, 165.

Working for the Profession, 112.

### ORIGINAL PAPERS.

A Plea for the Adoption of Oral Prophylaxis, by Grace P. Rogers, 597.

Avoiding Accidents and Failures in Anesthesia, By E. Loeffler, 17.

Business in General Practice, by George F. Burke, 6.

Compressed Air, George Zederbaum, 115.

Controlling a Hyposensitive Palate when Taking Impressions, by A. E. Franklin, 398.

Criticism of Prophylaxis, by C. M. Wright, 496.

Filling Molars that Require Dentalization, by Dr. G. F. Hines, 281.

Fixed versus Removable Bridge Work, by Dr. Robert B. Howell, 167.

Gold Inlay, J. W. Lyons, 500.

Hemophilic and Secondary Hemorrhage by George Zederbaum, 275.

Little Helps, by George Zederbaum. 618.

Mal-occlusion of the Teeth and its Intimate Relation to Carious Teeth, F. E. Williams, 513.

Mal-occlusion of the Teeth, Cause and Correction, F. E. Williams, 182.

Method of Producing Perfect Joints in Gum Sections, R. E. Petty, 356.

Missing Teeth and their Relations to Orthodontia and Bridge Work, M. T. Watson, 437.

Necessity for Reform in Dental Education by D. D. Smith, 542.

Odontalgia, by Wm. D. McFadden. 620.

Oral Hygiene Excerpts, N. S. Hoff, 339.

Pathologic Conditions of the Third Molar, B. S. Sutherland, 191.

Pathology of Tooth Decay, R. W. Bunting, 221.

Practical Points in Filling Teeth, by H. P. Martin, 345.

Presidents Address, J. J. Green, 446.

Pressure Anesthesia, George Zederbaum, 80.

Professional Ethics, T. R. Buttrick, 59.

Proving the Articulation, Countour and Expression, G. H. Wilson, 382.

Review of Pyorrhoea Alveolaris Literature, G. C. Bowles,

Use of the Pyrometer in Fusing Porcelain, R. M. Pelton, 450.

Value of Dental Society, R. W. Morse, 284.

Why the So-called Gold Annealer Should be Used in Giving the Heat Treatment to Gold Foil, M. L. Ward, 388.

### REPRINTS.

Aluminum Plates, Geo. D. Sitherwood, 93.

Chemistry of Plaster of Paris, by W. J. Manning, 21.

Dentistry a Worthy Profession, S. H. Guilford, 399.

Funomia, R. B. Fuller, 21.

Gold Solder, S. H. Guilford, 458.

High Pressure Anesthesia, S. M. Weaver, 128.

Obtunding Dentine, Ellison Hillyer, 134.

Orthodontia Notes, W. J. Brady, 357.

Proper Light and Arrangement of the Dental Operating Room, by Geo. B. Sanford, 622.

Pyorrhoea Treatment, A New Feature, W. V.-B. Ames, 30.

Treatment of Putrescence, A. W. Harlan, 229.

What Does the Dental Profession Most Need, T. W. Brophy, 289.

### WITH OUR EDITORS.

Advancement and Conservatism of Energy, G. E. Hunt, 470.

A National Standard, H. Prinz, 195.

A Phase of Ethics, C. N. Johnson, 527.

A remedy for the Troubles in the National Dental Association, R. Ottolengui, 359.

Better Management of Large Dental Convention, J. N. Crouse, 410.

Colleges and State Boards, C. N. Johnson, 298.

Concentration, C. N. Johnson, 194.

Concerning Hobbies, E. C. Kirk, 238

Criticisms of Dental Colleges, T. J. Collins, 294

Crown or Not to Crown, C. N. Johnson, 24i5.

Dental Medicines, T. J. Collins, 295.

Dental Soceties, 299.

Dentists Rank, W. J. Brady, 463.

Dentistry for Public School Children in Germany, W. F. Litch, 466.

Duties of State Boards of Examiners J. N. Crouse, 529.

Educate the Press, L. P. Bethel, 249.

Elder Three Among Colleges, W. F. Litch, 364.

Failure of Congress to Pass the Army and Navy Dental Bills, W. F. Litch, 522.

Health of the Dentist, 201.

High Fees, 297.

How Should a Dentist Invest His Savings, S. H. Guilford, 246.

Investigate the Criticism, Geo W. Cook, 366.

Narrowing the Foreign Field for American Dentists, W. F. Litch 626.

National Dental Association Meeting Atlanta, R. Ottolengui, 532.

Our Creed, C. A. Kimball, 525.

Painless Excavating not an Open Question, W. G. Ebersole, 146.

Passing of the International Dental Journal, E. C. Kirk, 145.

Peddling Nostrums, A. E. Webster, 199,

Politics in Dental Societies, J. N. Crouse, 623.

Relief for San Francisco, C. N. Johnson, 296.

Respect for the Essayist, H. Prinz, 408.

Retrogression or Advancement, Which? L. P. Bethel, 141.

State Societies, J. N. Crouse, 251.

The Wheat and the Chaff, J. D. Patterson, 405.

What Will the Program Be? J. N. Crouse, 179.

Who Shall Fix the Standard, J. D. Patterson, 143.

Wood Alcohol and its Dangers, W. F. Litch, 241.

### BIBLIOGRAPHY.

Guilford's Orthodontia, 95.

International Dental Congress Transaction, 161.

Kirk's American Text Book of Operative Dentistry, 35.

Long's Dental Medicine, 34.

### OBITUARY.

M. H. Chappell, 107.

Charles C. Chittenden, 37.

D. W. Clancy, 36.

D. H. Crouse, 97.

F. S. Greaves, 108.

Oscar N. Heise, 322.

H. M. Lewis, 158.

C. W. Stainton, 432.

### INDENTS.

Aconite and Chloroform, 416.

Action of State Board Illegal, 257.

Adaptation of Partial Dentures, 39.

Adrenalin and Cocaine, 369, 209.

Air in Fillings, 473.

Alloying Crowns Prevented, 316.

Aluminum Solder, 99.

Alveolar Abscess, 370.

Alveolar Abscess Symptons, 149.

Alveolar Hemorrhage, 413

Alypin, 305, 478.

Amalgam, 45, 536.

Amalgam Dies, 370.

Amalgam Mixing, 536.

Amputating Root Ends, 258.

An Equitable Fee Basis, 422.

Anesthetic Trinity, 413.

Anesthetizing Pulp without Syring, 368.

Antiseptic and Anesthetic Paste,306

Antiseptic Liquid Soap, 100.

Antral Abscess Diagnosis, 100.

Antral Diseases, Causes, 105.

Antrum Infection by the Teeth. 634.

Atrophied Teeth, 156.

A Wasteful Lot, 316.

Bake, not Broil Porcelain, 304

Be a Roycrofter. 631.

Borax, Bubbling Prevented, 368.

Bragging Dental Society, 425.

Brooch Sterilizing, 39.

Calculus to Soften, 412.

Carbolic Acids in Cavity—Preparation, 535.

Case in Practice, 255.

Chloroform Death, 412.

Clay Pipe, 206.

Cleaning Teeth, 40.

Coating for Plaster Costs, 40.

Collect Bill, 308.

Construction of Properly Formed Crowns, 537.

Contouring a Bridge, 259.

Correct Bite, 309.

Cuspid or Canine, 154.

Davis Crown, Sitting Pin, 308.

Decayed Teeth, 416.

Decoloring Stained Instruments, 304

Decrease in Registration, 415.

Delay Extraction of Abscessed Teeth, 421.

Dental Education, Sensible Comment, 424.

Dental Art, 536.

Dental Caries and Osteoclasts, 306.

Dental Caries Prevented, 301.

Dental Styptic, 307.

Dentist and the Societies, 46.

Dentistry of Tin-Gold Fillings, 473.

Diffused Alveolar Abscess, 150.

Dishes for Acid Bottles, 307.

Double Step Hood, 420.

Duplication of Plaster Models, 45.

Eating Quickly, 259.

Edema from Infection, 153.

Effective Ligature, 312.

Errors in Practice, 101.

Extraction of Molar Tooth Restores Sanity, 633.

Facial Neuralgia, 101.

Failures, 635.

False Sympathy, 368.

Familiarity with Patients. 632.

Fees 634.

Fees and Ethics, 149.

Flasks for Duplicating Plaster Models, 99.

Gasoline Generators, 99.

General Anesthetics. 481.

Gold Filling with Cement Protection, 476.

Gold Inlay, 102, 208.

Gold Matrix to Prevent Fusing, 303.

Guarding the Cavo-incisol Edges of Inlays with Gold, 477.

Handling Sore Teeth, 311.

Hard Plaster Models, 304.

High Pressure Disensitizing, 43, 538.

Hot Air as a Pyorrhoea Treatment, 41

How a Dental College Looks to Some, 415.

Humanitarian Dentistry. 630.

Hydrogen Dioxide, 314, 535.

Immutability versus Progress, 479.

Impression Material, 418.

Impression and Dies for Root Stump 475.

Infected Third Molar Sockets, 371.

Ingredients of Tooth Powders, 371.

Inherited Obligation, 419.

Inlay Matrix Burnishing, 254.

Inlays a Gold Filling 304.

Investing Bridge Work, 311.

Irish, 41.

Is a Painless Dental Operation Profitable, 47.

Is a Painless Dental Operation Possible, 43.

Jenkins Porcelain, 420.

Local Anesthetic. 632.

Liquid Flux, 369.

Little Things in Dentistry, 26.

Making Gold Foil Cohesive, 100.

Matrix for Pouring Plaster Moulds, 535.

National Dental Law Wanted, 256.

Necessity for Prophylaxis, 258.

New Property of Aluminum, 314.

New Solvent for Gold. 630.

Nitrous Oxide Death, 303.

Office Coats, 306.

Organization, 417.

Oxidizing Agents in Dentifrices, 427.

Pain after Extraction, 42.

Plastic Fillings, Anchorage, 306.

Plate Retention, 584.

Penalty for Accident, 368.

Perfect Contact for High Pressure Needle, 40.

Practical, 313.

Preparation of Cavities with Nitrous Oxide 630.

Preparing Inlay Cavities, 207.

Preventing Caries, 309.

Preventing Cement Adhering Under Bridges, 305.

Printer's Blunder, 304.

Prophylaxis—What it Means, 103.

Protecting Porcelain During Soldering, 305.

Proximal Cavities, 307.

Pulp Capping, 414, 417.

Putrescent Canals, 413.

Putrescent Pulp in Diciduous Teeth. 42.

Pyorrhoea Treatment, 312.

Removable Porcelain Crown, 315.

Removal of Foreign Body from Oesophagus, 256.

Renewing Zinc, 254.

Repairing Spots in Pink Rubber, 307

Retaining Dentures, 305.

Replacing a Porcelain Facing, 101.

Restricting the Flow of Solder, 303.

Retention of Artificial Dentures, 585

Richmond Metal, 310.

Root Treatment, 306.

Rosin Soap on the Belt, 303.

Rubber Dam Adjustment, 155.

Rubber Excess, 309.

Rust Prevented, 304, 305,

Sanitary Bridge Work, 152.

Selecting Shade of Crown 303.

Setting Logan Crown, 536.

Setting Plaster, 39.

Separating Varnish, 368.

Sharpen the Lathe Cove, 40.

Silon Nitrate with Cement Fillings, 41.

Silon Stains, 206.

Silver Nitrate with Cement Fillings.

Sodium Dioxide Bleaching, 44.

Soft Solder Flux, 413.

Soldered Gold Crown from One Piece of Metal, 474.

Soldering Pliers, 414.

Spence Metal, 306.

Stano-Percha, 104.

Starting Gold Fillings with Cement, 255.

Sterilization of Cutting Instruments, 44, 257, 412.

Sterilizing Mouth Mirrors, 39.

Sterilizing the Mouth. 310,

Sterilizing Polishing Stones, 99.

Stovaine, 260.

Stuttering Cured by Correcting Malocclusion, 39.

Tempering Gold, 418.

Tin and Gold, Why Used, 474.

Tired Brain Prescription, 211.

Tooth Brush, care of, 214.

Topical Applications. 631.

Trade or Profession, 209.

Treatment of Erupting Teeth, 633

Tymophene, 414.

Useful Hints, 151.

Water versus Resine Impression, 313

Wax for Natural Bite Crown, 99.

Wire Loop, Clasp, 254.

Value of Dentistry, 311.

Vice Versa, 412.

**ANNOUNCEMENTS.**

Alkaloidal Clinic, 49.

American Dental Society of Europe, 48, 375.

American Orthodontia Society, 637.

Anesthesia Post Graduate School, 374.

Anniverary Banquet, 377.

Anniversary of St. Louis Dental Society, 432, 485

Army and Navy Dental Bills, 590.

Barnes University Dental Faculty, 161.

Banquet to Dr. G. V. Black, 589.

Bethel, Dr. L. P. Resigns, 319.

Central Michigan Dental Society 269.

Chappell, M. H. dead, 107.

Chicago College Alumni Clinic, 50.

Chicago Dental College Anniversary 636.

Columbus Dental Library, 485.

Columbus Ohio Dental Society, 376.

Dental Exhibit, 52.

Dental Hints, Discontinues Publication, 158.

Dental Review's New Cover, 51.

Dental Supply Houses Exhibit, 49 159.

Dentists Magazine, 51.

Detroit College of Medicine, Dental Department, 319.

Detroit Dental Society, 54, 161.

Faculty Resignation, 107.

Fatal Hemmorrage from Tooth Extraction, 50.

Federation Dentaire, 375.

Field, C. Kingsley, 318.

Fraternity Dinner, 213.

Free Dental Service for the Poor, 378

Freeman George M., Married, 108.

Gilbert, W. H., President, 216.

Gould's Dictionaries, 213.

Graduates in 1906, 484.

Greaves, F. S., dead, 108.

Harvard Dental Alumni Association 540.

Hettinger Bros., Dental Depot burned, 159.

History of Dentistry, 160.

Illinois State Board of Examiners, 540, 264.

Illinois State Dental Society, 319, 216.

Indiana State Board of Examiners, 264.

Indiana State Dental Association, 213.

Institute of Dental Pedagogics, 213, 589, 637.

Interstate Dental Fraternity, 267, 318

Iowa State Board of Examiners, 588

Jones, Dr. J. 317.

Kansas City Clinic, 214

Kentucky State Board of Examiners 269, 590.

Kentucky State Dental Association, 215, 266.

Law, Wm. G., 318.

Lewis, H. M., dead, 158.

Louisiana State Dental Society, 270.

McKellops, Gerold, dead, 538.

Miami Valley Dental Society, 265.

Michigan State Board of Examiners, 49, 540.

Michigan State Association, 270, 321, 430.

Missouri State Dental Association, 215, 376.

National Dental Association, 377.

National Dental Association Officers 538.

National Association of Examiners, 214.

National Association of Faculties, 267.

New Hampshire Dental Society, 50.

New Jersey State Board of Examiners, 212

New Jersey State Dental Society, 215

New York State Dental Society, 376

New York State Journal of Medicine 269.

No Portraits, 378.

Northern Ohio Dental Association, Officers, 540.

Oakman Charles H., 318.

Ohio College of Dental Surgery Commencement, 319.

Ohio Dental Law Constitutional, 108.

Ohio State Board of Examiners, 539, 216, 266.

Ohio State Dental Society Meeting, 589.

Ohio State Dental Society Officers, 54,

Orthodontia Society, a New One 159.

Patents, 159.

Pennsylvania College of Dental Surgery Anniversary, 266.

Public Dental Library, 374.

Quonehtacut Club, 107.

Rail Road Rates to Atlanta, 486.

Reciprocity in Iowa, 320.

Rochester, N. Y. Dispensary Report, 373.

San Francisco Dental Relief, 268, 321.

Segur, F. D., married, 50.

Sentenced to Prison, 52.

Somnoform Death, 539.

South Dakota Examining Board, 376.

Southern Dental Society, of New Jersey, 51.

Southern Nebraska Dental Society, 539, 636.

Southwestern Michigan Dental Society, 160.

St. Louis Dental Society, 318.

Swiss Odontological Society, 375.

Tennessee State Dental Association, 266.

Vanderbilt University Dental Clinic, 270.

Western Dental Journal, 158.

Will Stay out of Politics, 373.

Wisconsin State Board of Examiners, 53

Woman Dental Association, 308.

Worboys, Clare, G., dead, 37.

### AUTOGRAPHIC.

Allen, C. C., 476.

Ames, W. V-B., 30, 14, 631.

Anjubalt, F. H., 415.

Ashley, K. P., 260.

Arnold, L. H., 311.

Bawget, G. T., 255.

Bethel, L. P., 39, 141, 249.

Black, A. D., 370.

Black, G. V., 149, 151, 154, 473.

Bogue, E. A., 413.

Boom, H. H. 373-430.

Bowles, G. C., 329, 444.

Brady, W. J., 357, 463, 631.

Brinton, W. P., 414.

Brocket, C. A., 100, 256.

Brophy, T. W., 289.

Brown, G. G., 309.

Brownlee, Dr., 177.

Bunting, R. W., 221.

Burke, G. F., 6, 16.

Butterick, T. R., 59, 79, 91.

Byram, J. Q., 304.

Callahan, J. R., 474.

Capon, W. A., 333.

Chappell, M. H., 107.

Chilson, 150, 311.

Collins, T. J., 294, 295, 14.

Conroy, J. K., 415.

Cook, Geo., W., 141, 366, 233, 236.

Crosland, J. K., 104, 258.

Crouse, J. N., 197, 251, 410, 529.

Cryer, M. H., 634.

Davidson. C. W., 307.

Dais, H. E., 632.

Davis, H. E., 369.

Davis, W. C., 369.

Dickil, J. L., 18.

Dorr, P. P., 303.

Doughaday, A., 368.

Dunn, J. Austin, 156.

Easton, W. S., 254.

Ebersole, W. G., 146, 584, 631.

Ellis, W. H., 44.

Endelman, J., 584.

Fagan, F. H., 264.

Fernandes, E. M. S., 312.

Fleming, A. H., 102.

Frank, G. E., 99.

Franklin, A. E., 398.

Franklin, C. P., 212.

Franklin, F. W., 303.

Gabriel, W. M., 305.

Gallie, Don. M., 417.

Garhart, N. K., 125.

Gaslee, H. J., 137, 311.

Gould, H. P., 140.

Graham, D. W., 355.

Graham, F. C., 40.

Green, J. J., 446.

Grievers, C. J., 478.

Grover, W. R., 313.

Guilford, S. H., 246, 399, 458.

Hadley, G. E., 397.

Hall, J. S., 352.

Hamilton, H. L., 309.

Hampton, C., 89, 119.

Hardgrove, T. H., 413.

Harlan, A. W., 257, 229, 237.

Harper, W. E., 394.

Harrell, H. B., 538.

Haskell, L. P., 585.

Hayter, Mark, 536.

Hewitt, A. C., 413.

Hillyer, E., 134.

Hines, P. F., 281.

Hinman, T. P., 420.

Hoff, N. S., 14, 339, 348, 350, 441, 444.

Holt, F. W., 12.

Houchen, S. A., 101.

Howell, R. B., 167, 179.

Howard, F. E., 104.

Hoyt, H. W., 535.

Hunt, G. E., 470.

Jack, Louis, 416, 417.

Jackson, H. N., 536.

Jackson, W. H., 510, 512.

Johnson, C. N., 194, 245, 296, 298 527.

Kaptan, E. M., 39.

Keeler, H. S., 100.

Kessel, R. ,310.

Kimball, C. A., 525.

Kirk, E. C., 414.

Land, C. H., 279, 511.

Lanphear, O. E., 396.

Lawrence, F. B., 234, 237.

Leonard, N. C., 138.

Line, J. E., 317.

Litch, W. F., 241, 364, 466, 522.

Logan, H. G., 308.

Loeffler, E. T., 17, 87, 121, 369.

Long, E. H., 481,

Low, F. W., 236.

Lyons, J. W., 500, 512.

McDonald, F. W., 14, 178.

Manning, W. J., 25.

Marshall, Jno. S., 136.

Martin, H. P., 345, 349.

Merrick, J. B., 308.

Meyers, C. G., 43.

Moody, J. D., 423.

Morse, R. W., 284.

Nesbit, Dr., 77.

Newkirk, G. E., 315, 316.

Nicholson, G. 206.

Nicholson, J. H., 312.

Noble, C. C. 172.

Noyes, F. B., 308.

Nyman, J. E., 536, 304, 632, 634, 635.

Ottalengui, R., 359, 532.

Patterson, J. D., 45, 143, 405.

Payson, W. S., 307.

Pelton, R. M., 450.

Petty, R. E., 356.

Platt, F. L., 47, 139.

Price, W. A., 43, 47.

Prinz, H., 195, 408.

Prothers, J. H., 536.

Register, H. C., 310.

Rhein, M. L., 138.

Rice, O. P., 209.

Richardson, M. H., 17.

Roach F. E., 153.

Roberson, W. A., 633.

Rogers, P. Grace, 597.

Rohland, Dr., 481.

Root, J. P., 310.

Sanford, Geo. B.,

Sauer, W. E., M. D., 261.

Schamberg, M. D., 258.

Schooley, H. M., 475.

Shattuck, G. S., 76.

Sitherwood, G. D., 93.

Smith, D. D., 542

Spalding, E. B., 178, 443, 349, 350, 355.

Sprague, H. A., 396.

Stalte, H., 100, 107.

Starr, A. R., 138.

Steel, J. F., 40.

Straith, S. 70.

Sutherland, B. S., 191.

Taggart, W. H., 209.

Taylor, L. C., 476.

Thompson, J. M., 16, 68, 90, 173,179 443, 353.

Thorpe, B. L., 403.

Truitt, G. E., 535.

Truman, W. H., 424.

Tuller, R. B., 40, 21, 44.

Walkerin, H. N., 255.

Ward, M. L., 388, 398.

Ward, R. J., M. D., 256.

Watkins, G. B., 11, 175.

Watson, M. T., 351, 437, 445.

Vaughan, Z. F., 418.

Weaver, S. M., 128.

Weber, H. L., 100.

Webster, A. E., 199.

Wedelstadt, E. K., 156.

White, O. M., 441, 521.

Whitefield, G. W., 305.

Whitslar, W. H., 412.

Williams, J. Leon, 39.

Williams, F. E., 182, 513.

Willman, A. C., 152.

Wilson, G. H., 155, 382, 588.

Winn, Jas. W., 371.

Wood, G. P., 15, 77, 176, 178, 444.

Worboys, C. H., 396, 508.

Zederbaum, Geo., 80, 91, 107, 122 275.

Ziegler, D. H., 630.

### ANNOUNCEMENTS.

AMERICAN DENTAL SOCIETY OF EUROPE.—The next meeting of the American Dental Society of Europe will be held at Berlin, Germany, the 1st, 2d, 3d and 4th of August,

1906. A most cordial invitation is extended to members of the profession to be present. An interesting program is already assured and it is hoped to make this one of the most interesting and largely attended meetings in the history of the society.

GEORGE O. WEBSTER, *Hon Sec'y*,
Pariser Platz, I, Berlin. Germany.

—✦—

MICHIGAN BOARD OF DENTAL EXAMINERS.—At the last regular meeting of the Michigan State Board of Examiners in Dentistry, held at Ann Arbor, October 31 to November 4, the following officers were elected to serve for the ensuing year: President, Walter C. McKinney, Saginaw; Treasurer, C. H. Oakman, Detroit; Secretary, Albert L. LeGro, Three Rivers.

Reciprocity with Oklahoma was ratified. Michigan now interchanges with Canadian Northwest Territories, New Jersey and Oklahoma. Next meeting will be held at Detroit in May, 1906.

DENTAL SUPPLY MANUFACTURER'S EXHIBIT.—The Ransom and Randolph Co. again entertained the dealers and manufacturers, December 12th to 14th. All the prominent manufacturers were present and a large number of dentists visited the exhibits and parted with their hard earned coin for the many new and attractive appliances which they saw and could not resist buying. We understand that a series of these exhibits are planned to cover the entire country. The next will be held in Chicago March, 27 to 30th, in the Auditorium Hall.

—✦—

THE ALKALOIDAL CLINIC.—With the burning of their factory, the entire publishing plant of this excellent medical journal was destroyed. The editor and publishers have determined to change its name to *American Journal of Clinical Medicine,* and hope to issue soon with a bettei magazine in every way. The editorial force will be augmented and an earnest effort will be made to print all that is best in the realm of medicine and its literature.

—✦—

CHICAGO COLLEGE ALUMNI CLINIC.—The eleventh annual clinic will be held January 17th and 18th, at the College. The entertainment will be papers, clinics and a banquet. All alumni are requested to attend.

RUDOLPH BECK, *Pres.*,
Stewart Building, Chicago.

—✦—

THE NEW HAMPSHIRE DENTAL SOCIETY.—The next session of this society will be held next May at the Pemigewassett House, Plymouth, N. H. This is at the gateway into the White Mountains, and will be an excellent place to go for a complete rest. Write to

F. F. FISHER, *Sec'y,*
Manchester, N. H.

—✦—

MARRIED.—Dr. F. D. Segur, of Muskogee, Indian Territory, was married December 16th, 1905, to Miss Myrtle Baker. The REGISTER congratulates the happy couple.

—✦—

FATAL HEMORRHAGE FROM TOOTH EXTRACTION.—The newspapers tell of the death of United States Senator Mitchell, of Oregon, from persistent hemorrhage following the extraction of four teeth. He had been in poor health for some time from diabetes and had no strength to resist the shock from the operation and loss of blood, although everything was done to check the hemorrhage and sustain the patient.

—✦—

THE SOUTHERN DENTAL SOCIETY OF NEW JERSEY.—This energetic dental society has issued its program for the year's work. It meets each month and has one good essayist for each meeting. Dr. A. W. Harlan will contribute to the January paper on "Disinfection as Related to Water, Instruments and Soft Tissues." The meetings are held at Camden, the third Wednesday of each month.

—✦—

THE DENTAL REVIEW comes out this month with a new cover. The border is made up of small portraits of distinguished dentists and is indeed unique. With such an exposition the editor will need to hustle to make a journal that shall satisfy the expectations of his readers, but Johnson will do it, if talent and conscientious hard work fail not.

—✦—

THE DENTIST'S MAGAZINE.—This is the new Cleveland Dental Journal to which we made reference in our July issue. Owing to publication difficulties it could not be issued until December. Wouldn't it have been just as well to have waited until January and thus have the volume consecutive with the calendar year? This would have given time also to eliminate many typographical errors, and other accidents which almost inevitably attend the launching of a new venture. The journal is well designed and beautifully printed and must have cost lots of thought and money to develop. The editorial quartette seem to have succeeded in developing each his own department in an entirely satisfactory manner. The original contributions are good and the selected articles show discretion. The DENTAL REGISTER welcomes the new enterprise and wishes it prosperity and success in developing greater interest in professional literature.

—✦—

DENTAL EXHIBIT.—On March 27, 28, 29 and 30, 1906, there will be held at the Auditorium, Chicago, the most complete exhibit of dental goods and appliances ever attempted. There will be no papers but continuous demonstrations in nearly every line of denstitry will be numerous. This is an exhibit by the manufacturers for the dentists and all are invited to attend. No dues or fees.

—✦—

SENTENCED TO PRISON.—Convicted of forging and selling dental certificates admitting incompetent students to practice, Dr. Jacob H. Smyser, was sentenced December 7th by Judge Smyth to pay a $300 fine and to the penitentiary under the indeterminate act.

Dr. Smyser was secretary of the State Board of Dental Examiners under Governor Tanner, and used his office, according to the evidence, to defraud the State. He was assisted by Edward Flynn, who was formerly an investigator employed by the dental board, who also was sentenced to the penitentiary and to pay a $300 fine.

The two men were indicted in 1901 for conspiracy to defraud the State, a year after Dr. Smyser had resigned from the board. The discovery was made that a large number of dental students, who had not spent the requisite number of years at a dental college, were securing certificates. Several of the incompetent students located in Germany.

Their incompetency attracted the attention of that government and complaint was made to the Illinois authorities against sending the products of "diploma mills" there.

The investigation which followed showed that Dr. Henry Messenger, 4712 South Ashland Avenue, had been reported to the State Board by Flynn as not possessing a license or certificate. Dr. Messenger was able to show that he had paid Flynn, as the agent, $10 for a temporary license, and had followed this up by a payment of $150 for a permanent license, which was recorded. There was evidence that the record had been erased from the books of the county clerk.

It was brought out during the trial that Dr. Smyser would send to the other members of the board, who lived in the southern part of the State, blank licenses and they would sign them for his use, giving him the authority to distribute them. It was shown that the license to Messenger was issued in December, 1900, three months after Dr. Smyser had resigned as secretary.—*American Dental Journal,*

—✦—

MEETING OF WISCONSIN STATE BOARD OF DENTAL EXAMINERS.—The next meeting of the Wisconsin State Board of Dental Examiners for examination of candidates for license to practice dentistry in Wisconsin will be held in Milwaukee, January 29, 1906, at the Hotel Phister.

Application must be made to the Secretary fifteen days before examination. The candidate must be a graduate of a reputable dental college, or have been engaged in the reputable practice of dentistry consecutively for four years, or an apprentice to a dentist engaged in the reputable practice of dentistry for five years. For further particulars apply to

J. J. WRIGHT, *Sec'y,*
1218 Wells Bldg., Milwaukee, Wis.

OFFICERS OF THE OHIO STATE DENTAL SOCIETY.—At the fortieth annual convention the following officers were elected: President, Dr. H. L. Ambler, Cleveland, Ohio; First Vice-President, Dr. H. C. Brown, Columbus, Ohio; Second Vice-President, Dr. C. I. Keely, Hamilton, Ohio; Secretary, Dr. F. R. Chapman, Columbus, Ohio; Treasurer, Dr. Weston A. Price, Cleveland, Ohio; directors for three years, Dr. L. P. Bethel, Columbus, Ohio; Dr. J. R. Calahan, Cincinnati, Ohio; Dr. Henry Barnes, Cleveland, Ohio, and Dr. W. T. McLean, Cincinnati, Ohio.

DETROIT DENTAL SOCIETY.—The annual clinic of this society will be held in the Detroit Dental College, February 8th, 1906. There will be all day clinics and a lecture on cavity preparation by Dr. G. V. Black, and a lecture by Dr. J. Q. Byram on the selection of colors for inlays. In the evening there will be a banquet at the Fellowcraft Club. The following is a partial list of clinic:

Dr. Black, "Preparation of Cavities for Metallic Fillings;" Dr. Roach, Chicago, "Removable Crown and Bridge Work;" Dr. Byram, Indianapolis, clinics and lecture on "Selection of Colors and Application of Porcelain for Inlays;" Dr. Worboys, Albion, Mich, "Jacket Crown and Gold Inlay;" Dr. D. N. Graham, Detroit, "Surgical Clinic;" Dr. Bowles, Detroit, "Gold Inlay;" Dr. H. Kreit, Detroit, "Continuous Gum;" Dr. Ames, Chicago, "Mixing of Cement and Shading of Colors;" Dr. H. C. Raymond, Detroit, "Preparation of Labial Cavities for Porcelain Inlay;" Drs. E. B. Spaulding, G. P. Rogers, G. P. Wood and N. S. Hoff, "Demonstrations of Methods Used in Oral Prophylaxis;" Dr. Frumviller, Detroit, "Lingual Attachments for Bridge Work;" Drs. White and Watson, Detroit, Dr. Abel, Toledo, "Orthodontia;" Dr. F. Logan, Detroit, Dr. Howell, Ann Arbor, "Bridge Work;" Dr. M. L. Ward, Ann Arbor, "Alloys."

